# Laser therapy in the treatment of feline sporotrichosis: A case series

**DOI:** 10.29374/2527-2179.bjvm005822

**Published:** 2023-04-25

**Authors:** Daniella Souther Carvalho Ribeiro, Luana Jordão Machado, Jéssica Gomes Pereira, Andrea Regina de Souza Baptista, Elisabeth Martins da Silva da Rocha

**Affiliations:** 1 Veterinarian, MSc. Programa de Pós-graduação em Microbiologia e Parasitologia Aplicadas (PPGMPA),Departamento de Microbiologia e Parasitologia,Instituto Biomédico, Universidade Federal Fluminense (UFF)- Niterói, RJ, Brazil.; 2 Veterinarian, Autonomous, Dermopet, Angra dos Reis, RJ. Brazil.; 3 Biologist, Instituto de Biologia , UFF. Niterói, RJ, Brazil; 4 Biologist, DSc., Departamento de Microbiologia e Parasitologia (MIP), Instituto Biomédico. UFF. Niterói, RJ, Brazil; 5 Veterinarian, DSc., Departamento de Microbiologia e Parasitologia (MIP), Instituto Biomédico. Universidade Federal Fluminense (UFF). Niterói, RJ, Brazil.

**Keywords:** *Sporothrix brasiliensis*, mycosis, alternative therapy, laser, *Sporothrix brasiliensis*, micose, terapia alternativa, laser

## Abstract

Sporotrichosis is the most prevalent subcutaneous mycosis in Latin America and is an important zoonosis in expansion throughout all the brazilian territory. Domestic cats are highly susceptible to the disease and play an important role in the spread of the agent to both other animals and humans. *Sporothrix brasiliensis*, the predominant species in the country, has greater virulence and some isolates also showed resistance to azoles, the class of antifungals of choice for treatment. Because it is a long-duration treatment, of high cost, and oral use, sick animals are often abandoned, which contributes to the spread and permanence of the infection as an important public health problem. Therefore, new therapeutic alternatives or adjuncts to treatment with antifungals may contribute to combating this zoonotic agent. In this work we describe the result of the treatment with laser therapy of eight *Sporothrix* spp infected cats. Our findings show the efficacy of the laser treatment even in different clinical forms. This technique has the potential to decrease the time length and costs of conventional treatment as well as the improvement of the treatment results.

## Resumo

A esporotricose é a micose subcutânea de maior prevalência na América Latina e se apresenta como uma importante zoonose em expansão por todo território brasileiro. Os gatos apresentam grande suscetibilidade à doença e assumem importante papel na disseminação do agente tanto para outros animais quanto para humanos. O *Sporothrix brasiliensis*, espécie predominante no país, apresenta maior virulência e alguns isolados também apresentaram resistência aos azóis, classe de antifúngicos de escolha para o tratamento. Por ser um tratamento de longa duração, de alto custo e de apresentação de uso oral, é frequente o abandono dos animais doentes, o que contribui para a disseminação e permanência da infecção como um importante problema de saúde pública. Sendo assim, novas alternativas terapêuticas ou coadjuvantes do tratamento com antifúngicos podem contribuir para o enfrentamento deste agente zoonótico. Nesse trabalho descrevemos os resultados obtidos no tratamento com a laserterapia em oito casos de gatos infectados por *Sporothrix* spp. Nossos achados mostram a eficácia do tratamento com laser mesmo em diferentes formas clínicas. Essa técnica tem o potencial de diminuir o período de tempo e os custos do tratamento convencional assim como melhorar os resultados do tratamento.

## Introduction

Sporotrichosis is the most common subcutaneous or implantation mycosis in Latin America (Silva et al., 2007) and its agents are fungi of the genus *Sporothrix*. The expansion of epidemic in Brazil has been correlated with the emergence of the species *S. brasiliensis*. This species presents great virulence and spreading capacity, with expansion throughout Brazil and to other Latin American countries, including Argentina, Paraguay and Panama (Córdoba et al., 2018; García Duarte et al., 2017; Gremião et al., 2020; Rios et al., 2018). Cat-to-human and cat-to-cat transmissions occur through bites or scratches of sick animals (Barros et al., 2001).

Feline sporotrichosis can be presented as a single or as multiple cutaneous lesions. Worse than that, cats can develop a disseminated systemic disease.

The treatment of sick animals is a strategy for controlling the infections, preventing the spread of the infectious agent to new non-human animals or humans. Cats under systemic antifungal therapy, due to the consequent reduction in the fungal load, do not appear to play a key role in the transmission the fungal cycle. Therefore, the early treatment of feline sporotrichosis should be always performed (Miranda et al., 2018). The feline treatment is the main challenge to face the disease once the treatment is costly and of long duration. In addition, some cats may be refractory to treatment. This results in a high dropout rate of feline sporotrichosis treatment (Gremião et al., 2021). In addition, itraconazole remains the drug of choice for the treatment (Gremião et al., 2021). In contrast, an increasing number of itraconazole-sensitive strains over time was documented (Almeida-Paes et al., 2017; Borba-Santos et al., 2015). Thus, new adjunctive therapies to increase the antifungal treatment are necessary and can contribute to the containment of the epizootic and the zoonotic epidemic.

In this work we describe the results obtained in a series of clinically and laboratory diagnosed cases of feline sporotrichosis treated effectively using photodynamic therapy as monotherapy.

Since the 1980s, several *in vivo* and *in vitro* studies have been reported on the anti-inflammatory, anti-analgesic and bio-modulatory actions of low-level laser therapy. Among the bio-modulatory effects of phototherapy, or low-level laser, are the therapeutics of morpho differentiation and cell proliferation, tissue neoformation, revascularization, reduction of edema, greater cell regeneration, increased local microcirculation and vascular permeability (Henriques et al., 2010).

In veterinary medicine, low-level laser became a popular modality in the animal rehabilitation scenario, widely used for pain reduction, inflammation reduction and wound healing (Gross, 2014). Among the applications scenario, they include postoperative cases, osteoarthritis, pain treatment, soft tissue injuries and wounds, treatment of neurological disorders, in acupuncture points, in dermatological problems (Gross, 2014), in bone cicatrization (Del Carlo et al., 2007), in nerve tissue repair (Oliveira et al., 2012). It is important to note that, as in human medicine, in veterinary medicine, photodynamic therapy has been used to eliminate multiresistant microorganisms (Sellera et al., 2019).

## Cases reports

This case series is based on records of domestic cats treated at two veterinary clinics in the state of Rio de Janeiro, from October 2018 to September 2021, diagnosed with sporotrichosis confirmed by imprint cytology and isolation of the agent in culture. Rescued animals that showed no clinical improvement for at least one month of treatment with itraconazole were included. The animals had different clinical forms of the disease, ranging from cases with respiratory compromise to the disseminated cutaneous form. The collected data included gender, age, neutering, diagnostic, previous treatment including drug(s) used and its duration, and complementary tests. In four cases, the person responsible for the animal reported the use of antifungal treatment - itraconazole, 100mg/day, orally, without apparent improvement and difficulty in administering the oral medication.

**Figure 1:**
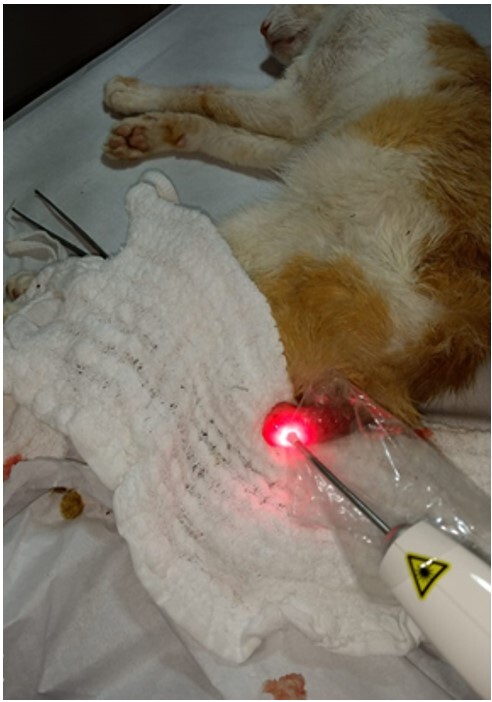
Application of laser therapy on the lesions of a feline with sporotrichosis. The pointer is applying the laser in a 90º angle, inside of lesion.

As these were semi-domiciled animals and were cared for by people with difficulties in administering oral medication or no clinical improvement after 30 days or even 60 days of antifungal treatment. Laser therapy was instituted with the suspension of the antifungals in most cases. Initially, the treatment started with the lesion asepsis using chlorhexidine 2% followed by drying with a gauze. These animals were submitted to laser therapy with red light by spot technique, respecting the distance of two centimeters between the application area. Applications were performed on the edge and inside the lesion, with the tip touching the wound at 90 degrees. By using this method, both the edges and the center of the lesion received irradiation aiming to induce tissue scarring and death of the fungus. Figure 1 shows a red-light application in a patient with a ulcerative lesion. The device used was Therapy (DMC) model.

The intensity of light energy, in joules (J), and the number of sessions in the prescribed protocol varied depending on the case presented and the response of each animal to the treatment. The protocols varied with applications between 1 and 6 Joules and between four and eight sessions (Table 1).

**Table 1:** Data regarding age, gender, previous treatments, clinic, and relapse of each feline sporotrichosis case.

	Age	Gender	Clinic	Previous treatment	Laser therapy	Post laser therapy treatment	Relapse
Case 1	3 y	Female	Ulcerated, necrotic and exudative lesion located in the nasal plane.	Itraconazole (100mg/day, orally), with no improvement.	4J, red laser, 4 sessions.	-	Monitored for 18 months, with no signs of relapse.
Case 2	10 y	Male	Ulcerated lesion in the dorsal region. Dehydrated, altered liver enzymes.	No specific treatment for sporotrichosis. In use of topical and oral antibiotic and anti-inflammatory drugs, no improvement.	1J, red laser, 4 sessions.	-	Monitored for 24 months, with no signs of relapse.
Case 3	1 y	Female	Crusted and ulcerated localized lesion in the dorsal region.	No previous treatment.	1J, red laser, 4 sessions.	Itraconazole 100mg/day orally for 30 days.	Monitored for 24 months without relapse.
Case 4	8 y	Male	Lesion located on the face.	No previous treatment.	1J, red laser. 4 sessions.	-	Monitored for 19 months without relapse.
Case 5	3 m	Female	Nodular, non-ulcerative lesion in the nasal plane. Nosebleeds when sneezing.	Itraconazole (50 mg/day, orally) for 30 days. Another 30 days with a dose of 100mg/day, at the tutor's own expense.	6J, red laser, 6 sessions.	Itraconazole 100mg/day orally for 30 days.	Monitored for 24 months without relapse.
Case 6	2 y	Male	Ulcerated lesions on the left ear, on the fore and hind limbs and on the tail.	Itraconazole 100mg/day orally for 60 days	Ear and limbs: 6J, red laser, 4 sessions. Tail: 6J, red laser, 6 sessions; 4 more sessions of 2J, red laser. Total of 10 sessions.	Itraconazole 100mg/day orally for 30 days.	Monitored for more than 10 months, relapse.
Case 7	3 y	Male	Disseminated sporotrichosis with ulcerated lesions on the face and limbs.	No previous treatment.	Methylene blue (0.01% in gel) 5 min plus 6J red laser (PDT). 2 more 2J red laser sessions.	Itraconazole 100mg/day orally, for 30 days.	Monitored for 12 months, no relapse.
Case 8	4 y	Male	Initial lesion in the scrotum. an increase in the nasal plane and constant sneezing. Vomiting episodes. Altered liver enzymes.	Itraconazole 100 mg/day orally and castration with ablation of the scrotum.	4J, red laser, 8 sessions. Plus itraconazole 100mg/day orally.	Itraconazole 100mg/day, orally, for 30 days.	Monitored for 24 months without relapse.

Y: Years old; m: months old; J: joule; PDT: Photodynamic Therapy.

The laser application does not require any use of sedatives on the animal since it is not painful. However, in some reported cases, the animal needed sedation in the initial sessions. This was done by using xylazine (0.5 mg/Kg) associated with cetamine (10mg/Kg) subcutaneously.

In some cases, new cytologies of the lesions were made between treatment sessions to verify if the therapy affected the fungal load. Laser therapy was instituted until complete healing of the disease. The identification of complete healing in the feline sporotrichosis is clinical, that is, when all clinical signs have completely disappeared (Gremião et al., 2015). In five cases, the use of itraconazole (100mg/SID) was prescribed for 30 days. The animals were monitored, most on average after a year or more post treatment. Table 1 shows the different protocols adopted in each case.

### Case 1

**Figure 2:**
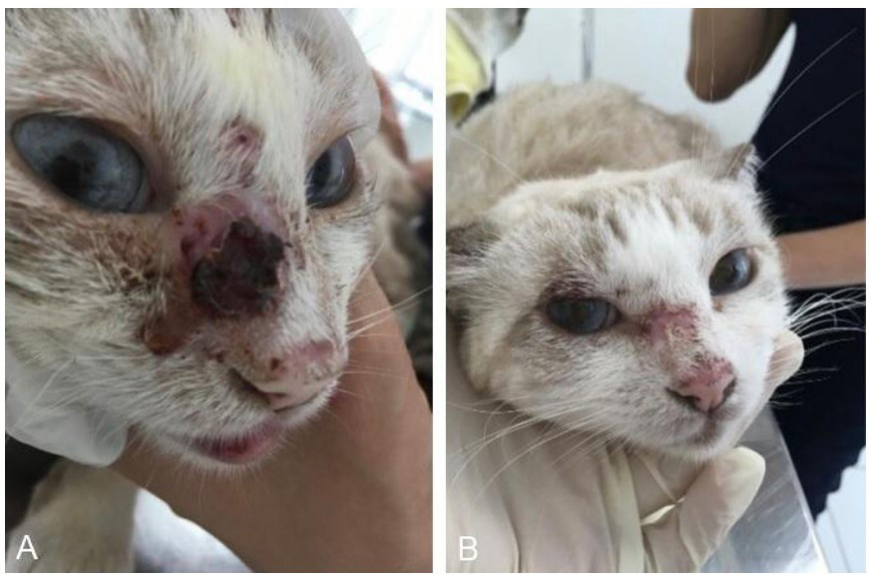
Evolution of lesions after photodynamic therapy.

Spayed, domestic short hair (DSH) semi-domiciled three years old, female presented an ulcerative, necrotic and exudative lesion located in the nasal plane (Figure 2A). According to the person responsible for the animal, she had been treated with oral itraconazole (100 mg/day), once a day, for 30 days, without any improvement. Due to the difficulty in administering the reported medication, the use of laser therapy was indicated along with the suspension of the antifungal. A weekly laser therapy session was performed with precise applications of 4J of red light.

(A) Female feline with sporotrichosis presenting an ulcerative, necrotic and exudative lesion located in the nasal plane before laser therapy; (B) Healed lesion after four laser therapy sessions.

The patient's evolution became evident from the second session onwards, with a reduction in the signs of ulceration and approximation of the edges, already showing a healing process indicated by granulation tissue (Figure 2B). It is worth mentioning that four sessions were enough for the remission of the lesion. After wound healing, the animal was monitored for 18 months, with no signs of lesion reoccurrence.

### Case 2

**Figure 3:**
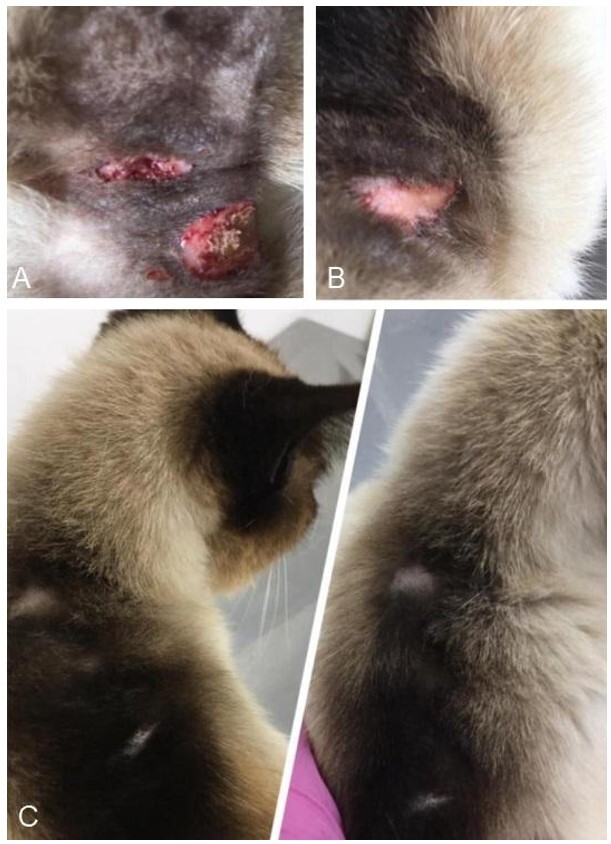
Evolution of lesions after photodynamic therapy (second case).

Spayed, DSH, semi-domiciled, ten years old male, with an ulcerative lesion in the dorsal region (Figure 3A). According to the person responsible for the animal, the feline was not undergoing any specific treatment for sporotrichosis but in the presence of antibiotic and topical and oral anti-inflammatory drugs, without improvement. Weight loss and apathy were also reported. In addition to confirming sporotrichosis in laboratory diagnosis, a physical examination showed dehydration signs and hematological tests (hemogram, biochemistry, FIV/FeLV tests) were also performed. Alterations were found in the liver aminotransferases, both AST (aspartate aminotransferase) and ALT (alanine aminotransferase), which were above the animal reference levels. Thus, laser therapy was started to treat sporotrichosis while preserving the liver functions because of the alterations found in the liver profile, low body score and signs of apathy.

(A) Ulcerative lesion in the dorsal region before laser therapy; (B) Lesions reduction in the third laser therapy session; (C) Complete healing of the lesions after the fourth laser therapy session.

A weekly laser therapy session was indicated. Four sessions were performed, with punctual applications at the edge of the lesion and inside was applied 1J, using red light. The patient's evolution became evident from the third session with wound reduction. The total healing of the lesions occurred after the fourth session when the patient was discharged. The animal was monitored for 24 months, and no manifestation of the disease was detected again. (Figure 3A, B, C).

### Case 3

**Figure 4:**
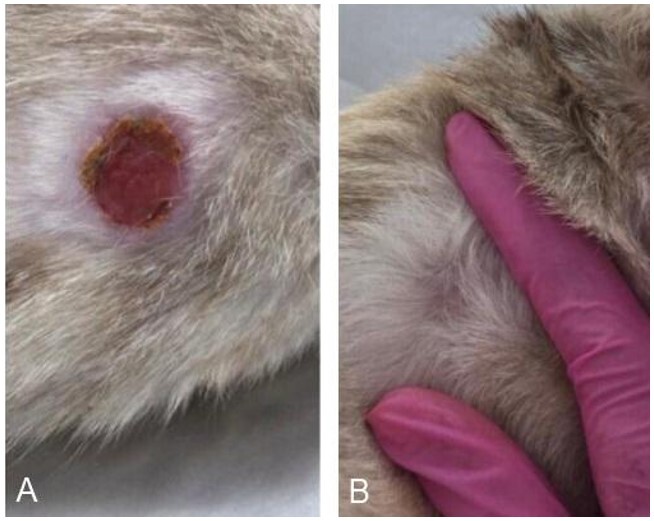
Evolution of lesions after photodynamic therapy (thrid case).

Female feline, DSH, spayed, semi-domiciled, one year old, with a localized crusted and ulcerated lesion in the dorsal region (Figure 4A). Laser therapy treatment was instituted based on a decision of the responsible for the animal. The treatment instituted was 1J laser therapy on the edge and interior of the lesion, performed in four sessions. This resulted in total wound healing (Figure 4B). After clinical cure, itraconazole 100mg/day was prescribed orally once a day for 30 days. After 24 months of monitoring, there was no relapse of the disease.

(A) Female feline with sporotrichosis and crusted ulcerative lesion in the dorsal region; (B) Total healing of the lesion after four sessions.

### Case 4

**Figure 5:**
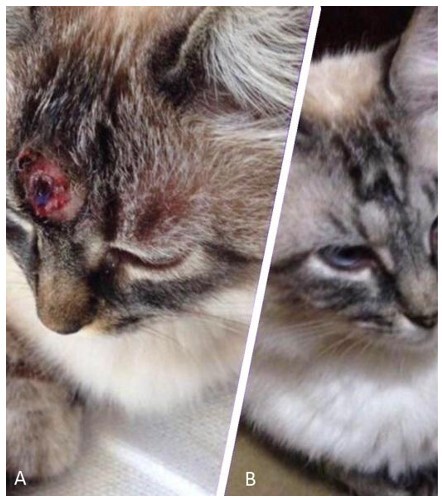
Evolution of lesions after photodynamic therapy (fourth case).

Spayed, DSH, semi-domiciled, eight years old, male presenting a lesion located on the face (Figure 5A). In this case, the use of oral itraconazole was not prescribed due to the difficulty in administering the medication to the animal reported by the person responsible for the animal. Therefore, laser therapy was instituted, with the use of 1J on the edge and inside the lesion and a space of 2cm between each point, performed in four sessions (one per week) with the observation of total wound healing (Figure 5B). The recommended azole was not prescribed in this case because of the aforementioned difficulty to administrate the medication. After clinical cure, a follow-up was carried out for 19 months, with no reoccurrence of the disease.

(A) Facial lesion; (B) Total healing of the lesion after four sessions of laser therapy.

### Case 5

**Figure 6:**
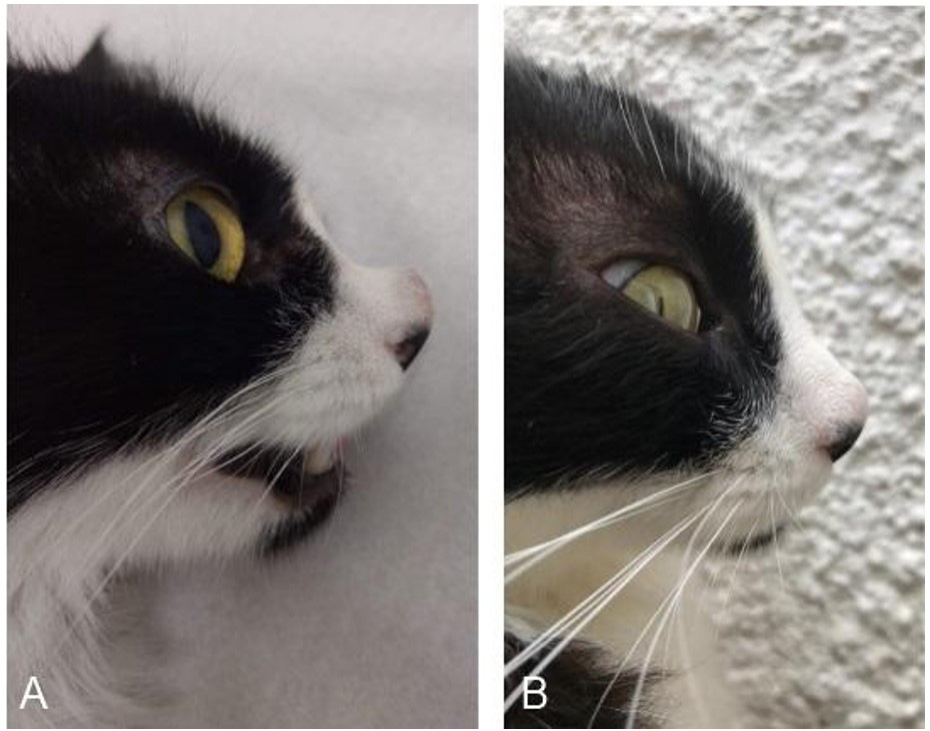
Evolution of lesions after photodynamic therapy (fifth case).

Spayed, DSH, semi-domiciled, female, three months old, was rescued presenting swelling in the nasal region. According to the person responsible for the animal, the effectiveness of oral treatment with itraconazole (50mg/day) for 30 days and for another 30 days with the dose of 100 mg/day, on its own, without a veterinary doctor’s prescription. During clinical examination, the animal presented a low body score, a non-ulcerative nodular lesion in the nasal plane (Figure 6A), nasal bleeding when sneezing. However, pulmonary and cardiac auscultation did not present any noteworthy alteration. During nasal cavity examination, the animal presented a hyperemic and swollen mucous, besides a discreet bleeding when exanimated. The person responsible for the animal was not able to perform any blood test to evaluate the hemogram and biochemistry. Since the treatment with itraconazole did not show any improvement in the patient’s condition, the use of antifungal was suspended and treatment using laser therapy was indicated.

(A) Evolution of laser therapy treatment in a female feline with sporotrichosis with a non-ulcerative nodular lesion in the nasal plane before treatment; (B) Total regression of the lesion after six treatment sessions.

There were six sessions with red laser, 6J per spot, weekly, and only in the first session the animal was sedated. Laser application was performed directly on the nose lesion skin, in a 90º angle. After the second session, the sneezing and nosebleeds ceased and the animal resumed normal feeding. A new cytology was performed after the third session and the result was negative.

Besides that, after the third session, the lesion had already reduced by 50% in its initial size and at the end of the treatment the reduction was complete (Figure 6B). With clinical cure, itraconazole (100mg/day), orally, was prescribed for 30 days. After this period, the animal returned for evaluation without any clinical signs. Weight gain was recorded. After 24 months of follow-up, there is still no relapse.

### Case 6

Spayed, DSH, estimated age of two years old, male, rescued with suspected disseminated cutaneous sporotrichosis, with ulcerative lesions on the left ear, on the fore and hind limbs and on the tail. The lesions bled easily when manipulated. Approximately 2/3 of the caudal vertebrae were exposed probably due to the chronicity of the infection and the virulence of the agent, being then amputated. The remaining 1/3, despite being completely skinless until the base of the tail, has been preserved.

At the time of consultation, the feline had a good body score, was active, with no changes in abdominal palpation or in cardiac and pulmonary auscultation. A hemogram was not performed due to a lack of financial conditions. The person responsible for the animal was administering itraconazole 100mg/day, orally, for 60 days, on their own (without a veterinary doctor's prescription), and reported that there was a slight improvement in the first 30 days but did not notice any improvement after this period.

**Figure 7:**
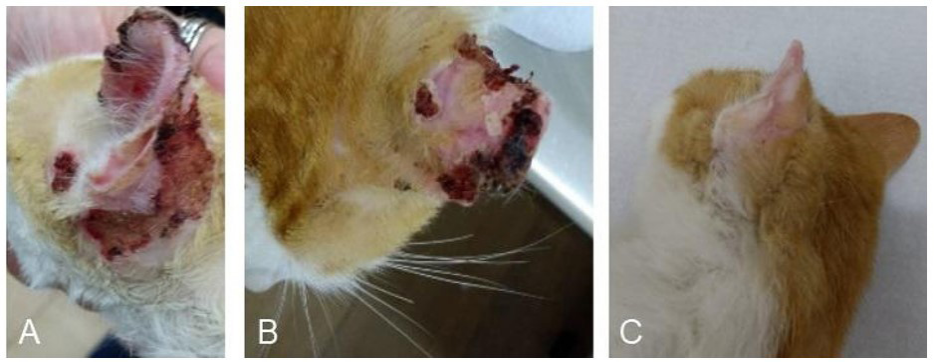
Evolution during treatment with laser therapy.

The use of itraconazole was suspended and laser therapy was started. Four sessions were performed with red laser, 6J per spot, weekly, for four weeks in the ear and limbs. In the tail, six sessions with 6J per spot and four sessions with 2J per spot were performed, resulting in a total of ten sessions. The animal was sedated in the first three sessions in which it presented great discomfort during manipulation. The animal showed a significant improvement soon after the second session, in which the lesions no longer bled so easily and began to show a healing process (Figure 7). Between the third and fourth sessions, ear cytology was performed and structures compatible with *Sporothrix* spp. were no longer observed. And between the sixth and seventh sessions, the cytology of the tail also no longer showed structures compatible with *Sporothrix* spp. The ear and limbs were completely healed after the fourth session, and the tail, after the eighth session, had scar tissue completely covering the limb. Itraconazole 100mg/day orally for 30 days was then prescribed. In follow-up for more than ten months, the animal showed no relapse of the disease.

(A) Ulcerative lesions on the left ear; (B) After the second session of laser therapy the lesion had an apparent improvement in the healing process; (C) After the fourth session, already completely healed.

### Case 7 - Photodynamic therapy combined with laser therapy

Male Feline, DSH, not neutered, estimated age of three years. At the time of the appointment, the animal presented disseminated cutaneous sporotrichosis with ulcerated lesions on the face and limbs, without changes in cardiac and pulmonary auscultation and also without changes in abdominal palpation. The blood test did not show any remarkable results.

The imprint cytology which confirmed the sporotrichosis diagnosis also revealed a significant number of cocci structures compatible with bacteria. Due to a great number of bacteria identified in the cytology, the chosen treatment consists a combination of photodynamic therapy (PDT) and laser therapy with red light. The first session was initiated after the orchiectomy procedure with the animal sedated. For the PDT technique, methylene blue dye (0.01% in gel) was applied to the lesion and after five minutes red light was applied, 6J per spot. After ten days of PDT, all lesions were in an advanced healing process (80% of reepithelization of the initial area). Two more sessions of red laser were then performed, one week after using PDT, 2J per spot, with an interval of three days between them, just to accelerate healing. In these sessions, the sedation of the animal was not necessary.

**Figure 8:**
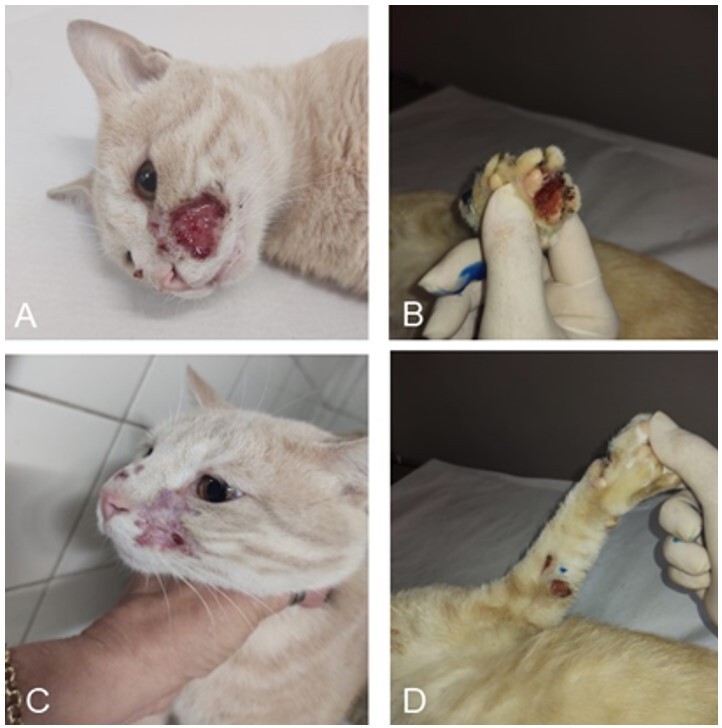
Feline presenting disseminated cutaneous sporotrichosis.

The presence of yeast-like structures compatible with *Sporothrix spp.* was not detected by cytology imprint in lesions after the very first session using red laser. The complete lesion scarring was observed 10 days after the second application of laser therapy with red light. The use of itraconazole 100mg/day orally was started days after clinical cure, and kept for 30 days. The animal remains without relapses after 12 months of follow-up (Figure 8).

(A) Ulcerative lesions on the face and (B) Ulcerative lesion on the footpad (both before treatment); (C) and (D) After ten days of photodynamic therapy (PDT) with the lesions in healing process after the first session.

### Case 8

**Figure 9:**
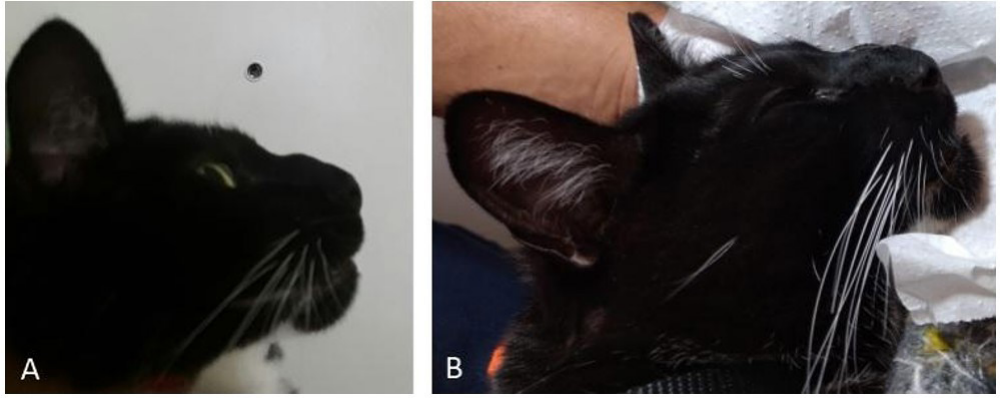
Feline presenting a tumor lesion in the nasal plane.

Male feline, DSH, four years old, was attended with a report of having already been diagnosed with sporotrichosis by another professional, with an initial lesion in the scrotum. Itraconazole 100 mg/day orally and castration with ablation of the scrotum were prescribed. After 30 days, the person responsible for the animal noticed an increase in the nasal plane (Figure 9A) and constant sneezing, and when contacting the veterinarian, they prescribed potassium iodide 25 mg/day orally in association with itraconazole. After 30 days of treatment the animal showed no improvement and started vomiting episodes. When taken to the clinic, the animal had an excellent body score, with no changes in the physical examination. Imprint cytology confirmed the diagnosis of sporotrichosis and a blood test was performed in which the hemogram showed no change. However, a biochemical test showed alterations in the ALT (440 IU/L), AST (184 IU/L) and FAL (398 IU/L), suggesting hepatic alteration. Treatment with laser therapy was then initiated.

(A) Before treatment with laser therapy; (B) Apparent regression of the lesion after the fifth session of laser therapy.

Eight sessions were performed with red laser, 4J per spot, weekly and only itraconazole 100mg/day orally was maintained. The first five sessions were performed with the animal sedated according to the previously described protocol, and the last three only with restraint.

During the fifth session, the lesion in the nasal plane showed significant regression (Figure 9B). It is important to note that a fine needle aspiration puncture was performed for a new cytology, in which structures compatible with *Sporothrix* spp were not observed. However, as the animal still had episodes of sneezing, the sessions were maintained until they ceased, which occurred after the eighth session,

Once clinical cure was achieved, itraconazole 100mg/day, orally, was maintained for another 30 days and there has been no relapse since then (about 24 months of follow-up).

## Discussion

In this work, we described eight cases of sporotrichosis treated with laser therapy and presenting successful results. Itraconazole remains as the drug of choice for treatment and its effectiveness as a monotherapy has been recommended even when associated with potassium iodate in cases of compromised mucosae (Gremião et al., 2021). Because it is used daily, for a long period, and orally, the risks of zoonotic transmission are higher, which makes it difficult to treat sick animals. These limitations of treatment with oral antifungal agents result in high rates of non-adherence to treatment and abandonment of sick animals (Gremião et al., 2021). The reported cases here were of animals rescued many times by protectors who could not afford the costs of the recommended treatment. The oral form of administration is a general complaint from anyone who is responsible for a feline and the difficulty in handling the animals was well reported by them in cases 1, 3 and 4. In other cases, the person responsible for the animal did not notice any improvement in the animal’s condition when treated with azole monotherapy (cases 5 and 6). Cases of therapeutic failure have been reported before in the conventional protocols and seem to be common in feline sporotrichosis (Reis et al., 2016). Non-responsive cases may occur especially with animals showing multiple lesions and/or mucosal lesions and showing respiratory symptoms (Gremião et al., 2015; Pereira et al., 2010). In general, lesions in the nasal region are difficult to treat, and depending on the severity of the lesions (severe pyogramulomatous inflammatory infiltrate, high fungal load and large mucosal lesions) can impair their healing process (Gremião et al., 2015, 2021). In cases of sporotrichosis with mucosal involvement, mainly nasal with respiratory signs that can lead to decreased appetite, anorexia and worsening of the general condition of the animal, the recommended effective treatment is the association of itraconazole with potassium iodide (Gremião et al., 2021). In case 8, the animal was being treated with the combination of itraconazole and potassium iodide without improvement and with signs of toxicity such as vomiting. After hemogram and verification of liver enzyme levels, signs of liver changes were observed. According to the recently published feline sporotrichosis therapeutic management guide, monitoring of liver conditions is advisable (Gremião et al., 2021).

Here, we present three cases with granulomatous or ulcerated lesions of the nasal mirror and the successful use of laser therapy with clinical cure and without remission of the disease (cases 1, 5 and 8). For cases unresponsive to antifungal drug therapy and cases with signs of liver toxicity, laser therapy may be an efficient alternative according to our reports.

It is important to note that a follow-up of the fungal load was performed in some cases using imprint cytology. In case 6, for example, which presented a clinical form of disseminated cutaneous sporotrichosis, new cytology of some lesions between the third and fourth sessions of the therapy were negative, and it remained like this throughout the treatment. A negative cytology was observed in case 7 after using PDT and another laser therapy session with red light 10 days after sporotrichosis diagnosis. In this case, the PDT technique and red light were combined due to the abundant presence of bacteria observed through cytology test. The PDT technique may be an option to increase the effects of laser therapy with red light in sporotrichosis cases associated with bacterial infection. The PDT has been proved to be quite efficient in several cases of bacterial infections, including species that are multidrug-resistant to antimicrobials (St Denis et al., 2011)

The results so far demonstrated the fungicidal potential of laser therapy and how this treatment acts on the fungal load of the lesions, which may consequently contribute for reducing the zoonotic potential of the infection, especially because no one who is responsible for an animal has to deal directly with the animal. In fact, the sensitivity of microorganisms, especially *Sporothrix spp.*, to the heat generated by red laser light in a murine model has been reported (Guirado et al., 2018). Laser therapy in weekly sessions, in most of the cases presented here, did not require sedation of the animals, or at least not in all sessions, proving to be a feasible technique in the domestic feline medical clinic.

Another advantage of laser therapy lies in its duration when compared to conventional antifungal treatments. The minimal duration was 30 days (patients 1 to 4 and patient 7) and a maximum duration of 70 days (case 6).

A relapse after clinical cure may occur, which demonstrates the possibility of reactivation of the lesions despite the end of treatment (Gremião et al., 2015). After clinical cure was confirmed, for some animals in the cases described here, it was possible to prescribe itraconazole for another 30 days as recommended, while for others was impossible to administrate the drug, making this protocol impossible. Even so, it is important to emphasize that the animals were monitored for a long period of time and none of them showed remission of the disease.

The established protocol of laser therapy is individualized for each patient and should be based on the clinical form and monitored according to the patient's response, and whenever possible, follow the recommended guidelines to avoid relapses such as maintaining itraconazole for at least 30 days (Gremião et al., 2021).

The use of alternative therapies or efficient adjuvants that reduce the time of use of oral medication represents an important tool for the treatment of the disease and the confrontation of this epidemic zoonosis, as the data presented here with the use of laser therapy.

## Conclusion

The therapy with red laser proved to be effective for treating feline sporotrichosis. It showed good healing results both as monotherapy or ins association with itraconazole.
